# Oxygen and Ventilatory Output during Several Activities of Daily Living Performed by COPD Patients Stratified According to Disease Severity

**DOI:** 10.1371/journal.pone.0079727

**Published:** 2013-11-20

**Authors:** Antonio A. M. Castro, Elias F. Porto, Vinícius C. Iamonti, Gérson F. de Souza, Oliver A. Nascimento, José R. Jardim

**Affiliations:** 1 Pulmonary Rehabilitation Center of Federal University of São Paulo – Unifesp, São Paulo, São Paulo, Brazil; 2 Federal University of Pampa (Unipampa), Uruguaiana, Rio Grande do Sul, Brazil; 3 Adventist University, São Paulo, São Paulo, Brazil; 4 Nove de Julho University, São Paulo, São Paulo, Brazil; 5 Pulmonary Rehabilitation Center of Unifesp, São Paulo, São Paulo, Brazil; 6 Professor of Respiratory Diseases and Director of the Pulmonary Rehabilitation Center of Unifesp, São Paulo, São Paulo, Brazil; Universidad Europea de Madrid, Spain

## Abstract

**Objectives:**

To measure the oxygen and ventilatory output across all COPD stages performing 18 common ADL and identify the activities that present the highest metabolic and ventilatory output as well as to compare the energy expenditure within each disease severity.

**Materials and Methods:**

Metabolic (VO_2_ and VCO_2_), ventilatory (f and V_E_), cardiovascular (HR) and dyspnea (Borg score) variables were assessed in one hundred COPD patients during the completion of eighteen ADL grouped into four activities domains: rest, personal care, labor activities and efforts.

**Results:**

The activities with the highest proportional metabolic and ventilatory output (VO_2_/VO_2_max and VE/MVV) were walking with 2.5 Kg in each hand and walking with 5.0 Kg in one hand. Very severe patients presented the highest metabolic, ventilatory output and dyspnea than mild patients (p<0.05).

**Conclusions:**

COPD patients present an increased proportion of energy expenditure while performing activities of daily living. The activities that developed the highest metabolic and ventilatory output are the ones associated to upper and lower limbs movements combined. Very severe patients present the highest proportional estimated metabolic and ventilatory output and dyspnea. Activities of daily living are mainly limited by COPD’s reduced ventilatory reserve.

## Introduction

Chronic obstructive pulmonary disease (COPD) patients experience progressive difficulty to perform light (e.g. groceries shopping and domestic work) to heavy (e.g. long distance walking and playing sports) activities of daily living (ADL) [1]. Thus, every day ADL may become a high burden for COPD patients especially for the ones with severe stages of the disease [2].

In a classic study made by Gordon et.al [3], in 1958, which a cost energetic table was developed in healthy subjects, the oxygen uptake (VO_2_) and the carbon dioxide production (VCO_2_) were considered to be the most practical way to measure the metabolic and ventilatory requirements. Several years later, the metabolic and ventilatory uptake were measured by Velloso et al [4] and Chii Jeng et al. [5] in COPD patients during the completion of a few ADL. Velloso et. al. [4] were the first ones to show that moderate and severe COPD patients would perform simple ADLs using up to 55% and 60% of their maximal oxygen and ventilation uptake, respectively. Following Velloso’s finding, Chiin Jeng et.al. [5] found increased values of oxygen uptake in 27 COPD patients during ADL. Despite the impact these authors brought to the literature, three limitations were observed in their studies: 1) the small number of patients tested (nine in Velloso’s and 27 in Jeng’s study); 2) lack of assessment in all COPD stages; 3) few ADL studied (four in Velloso’s and five in Jeng’s study).

Recently, Vaes et.al. [6] measured the oxygen uptake in 97 moderate, severe and very severe COPD patients and in 20 healthy subjects performing five domestic ADLs. They showed that the patients used a significant higher proportion of their peak aerobic and ventilatory capacity than healthy subjects. Patients with GOLD stage IV, MRC dyspnea grade 5, or BODE score ≥ 6 points had the highest task-related oxygen uptake and dyspnea perception during the execution of proposed ADLs. However, the study was restricted to a few ADL.

Thus, the objectives of our study was to expand Vaes et.al.’s findings by measuring the oxygen and ventilatory output across all COPD stages performing 18 common ADL grouped into four domains (resting, personal care, labor activities and efforts). We hypothesized that the worse the disease severity, the higher the oxygen and ventilatory burden and dyspnea.

## Materials and Methods

This was a prospective study carried out at the Pulmonary Rehabilitation Center at the Federal University of São Paulo (Unifesp), Brazil. This study has been reviewed and approved by the Federal University of São Paulo (Unifesp) review board (registry number: 473/04).

 The inclusion criteria were: male and female COPD patients diagnosed according to the GOLD definition [7], minimum of 10 pack year history of smoking, expiratory volume in the first second/ forced vital capacity ratio (FEV1/FVC) <0.7 L, age over 40 years, no exacerbations during the past month, have signed the written consent form. 

 The exclusion criteria were: any muscular and skeletal disorders that might prevent adequate range of movements, obesity (BMI>30Kg/m^2^) (avoiding any associated restrictive ventilatory impairment), any neoplasia or unstable coronary disease, use of continuous oxygen therapy and participation in any physical training program.

**Figure 1:**
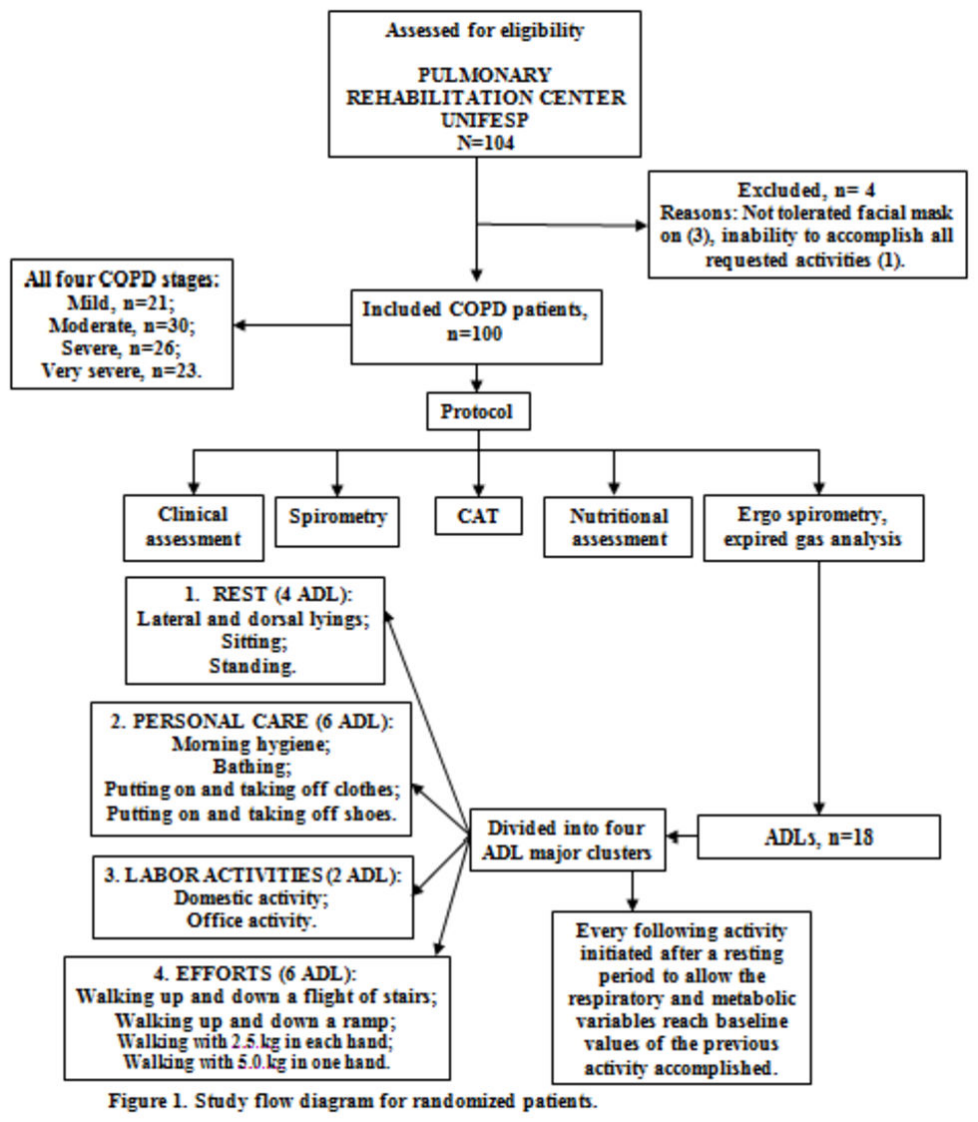
Flow diagram of the study design.

Patients completed medical examination, spirometry, the COPD Assessment Test (CAT) and a nutritional assessment. Patients were asked to perform 18 activities of daily living while the oxygen consumption (VO_2_), carbon dioxide production (VCO_2_) and pulmonary ventilation (V_E_) were continuously assessed by means of a metabolic system (Cosmed K4b^2^, Italy). Dyspnea, arterial blood pressure, respiratory and heart frequencies and oxygen pulse saturation were measured before and right after each activity. Patients had an adequate resting period prior to each activity, allowing all respiratory, metabolic and cardiac variables to return to baseline. The sequence of activities was the same for all patients (Figure 1). Every activity was performed just once following the same method of recent published studies [4,6].

### Measured activities

In order to standardize the movements patients would watch a video tape for each activity and were instructed to reproduce the movements as they were watching the video.

Due to the large number of activities tested, they were grouped into four clusters in order to facilitate the analysis: resting, personal care, labor activities and efforts (Figure 1). 

The activities were:

#### 1) Resting.

1) Dorsal and lateral lying positions: patients were comfortably placed in the dorsal and lateral lying positions on a bed for five minutes for each position. 2) Sitting: patients were comfortably placed in a back support ordinary chair while resting their feet on the floor for five minutes. 3) Standing: patients were placed in the standing position for five minutes.

#### 2) Personal care.

 4) Morning hygiene: brushing teeth for two minutes; washing face for two minutes; combing hair for one minute. 5) Bathing: patients performed movements as washing the head, thorax, abdomen and upper and lower limbs for five minutes.6) Putting on and taking off clothes and shoes: a surgical outfit and laced shoes were used. Patients initially put the clothes and shoes on and after enough resting period to ventilatory and metabolic variables return to baseline values they took off the outfit and shoes taking the time they usually spend to perform the activity.

#### 3) Labor activities.

7) Domestic activities: sweeping the floor for two minutes; storing pots weighting 1.5 Kg in upper and lower shelves for one minute; washing dishes, glasses and saucers for two minutes. 8) Office activities: writing in a sheet of paper for two minutes; answering the phone without any arm support for one minute; opening and closing drawers for one minute; moving paper sheets from one side to other of the desk for one minute. 9) Walking up and down a flight of stairs totaling 17 steps. Each step was 15cm high and 26 cm depth. The pace was set by the patients, according to their capacity to complete this activity. 10) Walking up and down a ramp of 13 meters long and 10% inclination. The pace was set by the patients, according to their capacity to complete this activity.

#### 4) Efforts.

11) Patients walked along a 25 m corridor for five minutes carrying 2.5 Kg in both hands for five minutes; after resting they repeated the test carrying 5 Kg in one hand for another five minutes.

### Spirometry

Three acceptable spirometry maneuvers (KoKo; Occupational Health Dynamics; Birmingham, AL, USA) were completed following American Thoracic Society/European Respiratory Society recommendations. Spirometry was repeated 15 min after the administration of a bronchodilator (albuterol 400 mcg); predicted values for FVC and FEV_1_ were calculated according to the third National Health and Nutrition Examination Survey [8,9]; the severity air flow limitations was classified according to Global Initiative for Chronic Obstructive Lung Disease [7]. 

### COPD assessment test (CAT)

Health related quality of life was assessed by the COPD Assessment Test (CAT) was developed to explore the general health status of COPD patients by measuring the impact of the disease and how it changes over time. CAT is simple to administer and aims to help clinicians manage a patient's COPD better [10,11]. 

### Measurement of metabolic, respiratory, and cardiac variables

The respiratory and metabolic variables were measured with the K4b2 metabolic system (Cosmed, Italy) in all activities performed. VO_2_ was measured as standard temperature and pressure, dry, in liters per minute and milliliter per kilograms per minute, and compared to the percentage of predicted VO_2_max values for patients with COPD. We estimated the VO_2_max according to the equation described by Carter et. al. [12] and previously used in our laboratory [4]: VO_2_max= 0.55 + (0.43 X FEV_1_). Oxygen consumption was described in ml^-1^kg^-1^min^-1^ in order to normalize the group for body weight. VE was measured and expressed in liters per minute. To compare the obtained VE to maximum voluntary ventilation (MVV), we estimated MVV values as FEV_1_ X 35 [13]. HR during exercise was analyzed with a pulse meter (Vantage XL; Polar; Kempele, Finland) with recordings at 5-s intervals. After the tests, the HR curves were stored in a computer. Maximum heart rate (HRmax) was evaluated in absolute terms (beats per minute) and as percentage of maximum predicted HR: HRmax = 220 – age [14].

Dyspnea was measured by means of the Borg scale and the pulse oxygen saturation was measured by a pulse oximeter (Nonin, USA) at the beginning and at the end of each activity performed.

### Nutritional assessment

#### Body mass index (BMI)

Body mass index was calculated as the weight/height^2^ ratio (kg/m^2^). Weight was measured on a calibrated scale (Filizola^®^, Brazil) and the height by an estadiometer with patients with no shoes on. Patients with BMI value under 22 kg/m^2^ were considered underweight, between 22 and 27 kg/m^2^ normal weight and over than 27 kg/m overweight [15] as it has been described for aged people and chronic disease. 

#### Bioimpedance

Bioimpedance was measured by means of electrodes placed on the right wrist and ankle of the patient comfortably placed on lying position for five minutes (RJL-101^®^, Comp Corp “software”, version 1.0) [15] and fat free mass (FFM) was calculated.

### Statistical analysis

The Kolmogorov-Smirnov test was used to ascertain the normality of data. We used the paired Student’s t test to analyze the differences between initial and final values for each activity accomplished. The ANOVA RM with the Bonferroni post-hoc correction test was used to assess the differences between anthropometric, pulmonary function, dyspnea, fatigue and respiratory and metabolic variables of each activity among all four COPD disease stages [16]. We considered a p < 0.05 as statistical significant.

### Sample size

Sample size calculation was based on the study primary objective, which was to analyze the energy expenditure of COPD patients performing activities of daily living. We calculated our sample size according to the formula E/S, where E= is the expected effect or the minimal difference and S= sample standard deviation. Since there are no COPD energy output table for life activities and expected minimal difference for these activities, we used for the calculation the mean difference values (final-initial) of these activities from a pilot sample in our lab. An E value of 0.7 L was found. The S value of 0.6 L for the same variable was taken from Vaes et al study [6]. Considering an α of 0.05 and a β of 0.2, 17 patients were necessary in each one of the four groups [16].

## Results

One hundred and four patients were invited to participate in the study; four of them were excluded from the protocol due to intolerance to the mask and inability to accomplish all requested activities (Figure 1).

One hundred patients of both genders and age over 40 years old were enrolled. Twenty one patients presented mild obstruction, 30 moderate, 26 severe and 23 very severe obstruction according to the GOLD criteria [7].

Patients presented a mean age of 65.1+10.3 years, and a very similar mean value for weight, height, BMI and FFM were found across all four stages of the disease. All COPD stages presented overweight and relative muscular mass preservation (table 1). Mild COPD patients had a better general health impact as compared to moderate, severe and very severe COPD patients as assessed by the CAT questionnaire (table 1).

**Table 1:** Anthropometric and pulmonary function characteristics of mild (n=21), moderate (n=30), severe (n=26) and very severe (n=23) COPD patients.

Variables/Severity	**Mild (n=21)**	**Moderate (n=30)**	Severe (n=26)	Very severe (n=23)	p
**Gender (M/F)**	**15/6**	**21/9**	**19/7**	**15/8**	**-**
**Age (years)**	**65.9±10.4**	67.8±8.7*	**65.0±9.2**	**59.4±11.8**	**0.007**
**Weight (Kg)**	**67.7±13.0**	**71.5±11.4**	**71.0±14.0**	**61.1±11.3**	**0.3**
**Height (m)**	**1.64±0.09**	**1.65±0.08**	**1.65±0.08**	**1.61±0.08**	**0.3**
**BMI (Kg/m^2^)**	**25.1±4.4**	**26.2±3.8**	**25.9±3.9**	**23.5±4.4**	**0.1**
**FFM (Kg/m^2^)**	**18.3±3.2**	**17.9±2.5**	**17.2±2.5**	**16.4±1.6**	**0.5**
**CAT**	**12.0±6.6^*#*^**	**18.6±9.5**	**19.9±8.8**	**25.3±8.8**	**0.01**
**FEV_1_/FVC (L)**	**0.64±0.05^*#*^**	**0.53±0.1^*&*^**	**0.40±0.05^$^**	**0.33±0.06**	**0.0001**
**CVF (L)**	**3.55±0.61^*#*^**	**2.98±0.79^*&*^**	**2.55±0.65^$^**	**1.97±0.53**	**0.0001**
**FVC (%)**	**109.0±14.4^*#*^**	**92.8±16.3^*&*^**	**75.2±12.9^$^**	**59.1±11.4**	**0.0001**
**FEV_1_ (L)**	**2.30±0.49^*#*^**	**1.56±0.44^*&*^**	**1.02±0.27^$^**	**0.64±0.13**	**0.0001**
**FEV_1_ (%)**	**91.0±8.6^*#*^**	**62.2±10.2^*&*^**	**38.7±4.7^$^**	**23.9±3.9**	**0.0001**

M= male; F=female; BMI= Body mass index; FFM= fat free mass; CAT= COPD assessment test; FVC= forced vital capacity; FEV_1_= expiratory volume in the first second.

*Moderate vs. very severe; **^*#*^**mild vs. moderate, severe and very severe; **^*&*^**moderate vs. severe and very severe; **^*$*^**severe vs. very severe.

Table 2 shows the oxygen consumption (ml^-1^Kg^-1^min^-1^) value at the end of the 18 activities performed. As expected, resting activities presented the lowest values of oxygen uptake and, on the other hand, activities of walking with 2.5 Kg in each hand and walking with 5.0 Kg in one hand presented the highest values (Table 2).

**Table 2:** Oxygen consumption (ml^-1^kg^-1^min^-1^) analysis in mild (n=21), moderate (n=30), severe (n=26) and very severe (n=23) COPD patients at the end of the 18 activities of daily living performed.

Activities/ Severity	**Mild (n=21)**	**Moderate (n=30)**	Severe (n=26)	Very severe (n=23)	p
**Lateral lying**	**3.4 (2.8-3.9)**	**2.6 (2.3-3.0)**	**2.8 (2.4-3.2)**	**3.0 (2.5-3.6)**	**0.3**
**Dorsal lying**	**3.1 (2.7-3.5)**	**3.3 (2.8-3.9)**	**2.9 (2.5-3.4)**	**3.2 (2.6-3.7)**	**0.7**
**Sitting**	**3.1 (2.6-3.7)**	**3.3 (2.8-3.7)**	**3.1 (2.6-3.6)**	**3.5 (2.9-4.0)**	**0.6**
**Standing**	**3.4 (2.9-3.9)**	**3.2 (2.9-3.5)**	**3.4 (2.9-3.9)**	**4.2 (3.6-4.8)**	**0.08**
**Morning hygiene**	**5.7 (4.0-7.4)**	**5.7 (4.8-6.5)**	**5.8 (4.9-6.7)**	**6.7 (5.6-7.8)**	**0.7**
**Bathing**	**8.5 (7.3-9.8)**	**8.1 (7.1-9.1)**	**8.0 (6.8-9.1)**	**9.1 (7.9-10.3)**	**0.2**
**Putting on clothes**	**8.2 (6.3-10.1)**	**8.4 (7.0-9.7)**	**7.8 (6.9-8.8)**	**8.9 (7.5-10.3)**	**0.7**
**Taking off clothes**	**8.4 (6.5-10.3)**	**8.5 (7.1-9.9)**	**7.0 (5.9-8.1)**	**6.4 (5.0-7.8)**	**0.1**
**Putting on shoes**	**8.6 (7.1-10.0)**	**8.3 (6.8-9.9)**	**6.3 (5.4-7.2)**	**8.7 (7.2-10.2)**	**0.06**
**Taking off shoes**	**7.8 (6.3-9.4)**	**7.8 (6.8-8.9)**	**7.6 (6.3-8.9)**	**7.0 (6.0-8.0)**	**0.7**
**Domestic activity**	**7.1 (6.2-8.0)**	**7.1 (6.1-8.2)**	**6.9 (6.1-7.8)**	**8.1 (7.1-9.1)**	**0.3**
**Office activity**	**5.9 (5.0-6.9)**	**5.7 (4.8-6.6)**	**5.6 (4.6-6.6)**	**5.6 (4.8-6.5)**	**0.9**
**Walking down a flight of stairs**	**7.7 (6.8-9.0)**	**7.7 (6.5-8.8)**	**6.1 (5.4-6.8)**	**6.5 (5.4-7.1)**	**0.06**
**Walking up a flight of stairs**	**8.8 (7.5-10.2)**	**8.5 (7.3-9.7)**	**7.1 (6.4-7.9)**	**7.2 (6.0-8.4)**	**0.06**
**Walking down a ramp**	**7.7 (6.5-9.0)**	**6.7 (5.9-7.5)**	**5.8 (5.0-6.6)**	**6.4 (5.5-7.3)**	**0.07**
**Walking up a ramp**	**7.8 (7.1-8.5)**	**7.7 (7.0-8.4)**	**7.3 (6.6-8.0)**	**6.7 (5.9-7.5)**	**0.1**
**Walking with 2.5 kg in each hand**	**12.2 (10.7-13.8)**	**12.3 (11.0-13.6)**	**12.5 (10.9-14.1)**	**12.1 (10.4-13.9)**	**0.8**
**Walking with 5.0 kg in one hand**	**13.1 (12.2-14.0)**	**13.2 (11.6-14.8)**	**12.7 (11.1-14.4)**	**12.1 (10.3-13.9)**	**0.4**

Mean (95% confidence interval).

**Figure 2:**
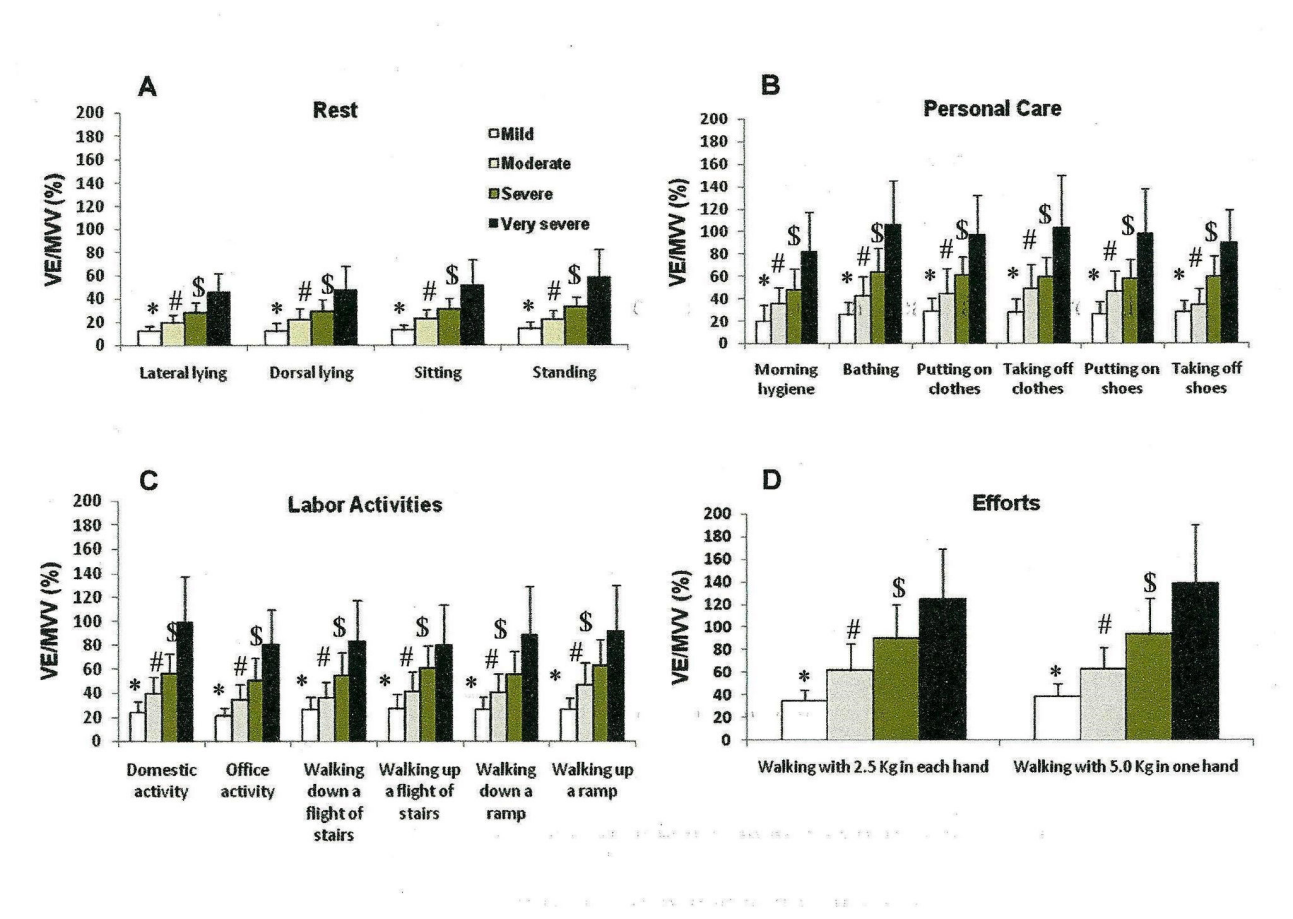
Proportional pulmonary ventilation/ maximal voluntary ventilation (VE/MVV %) ratio at the end of the group of resting activities (A)(lateral and dorsal lying, sitting and standing positions); of personal care (B) (morning hygiene, bathing, putting on and taking off clothes and shoes); of labor activities (C) (domestic and office activities, walking up and down a flight of stairs and ramp); and of efforts activities (D) (walking with 2.5 Kg in each hand and walking with 5.0 Kg in one hand) stratified by the GOLD spirometric classification (mild, moderate, severe and very severe). Mean±standard error. *p<0.01 - mild vs. moderate, severe and very severe; ^#^p<0.01 - moderate vs. severe and very severe; ^$^p<0.05 – severe vs. very severe. Short title: Proportional pulmonary ventilation/ maximal voluntary ventilation (VE/MVV %) ratio after the performed ADL.

**Figure 3:**
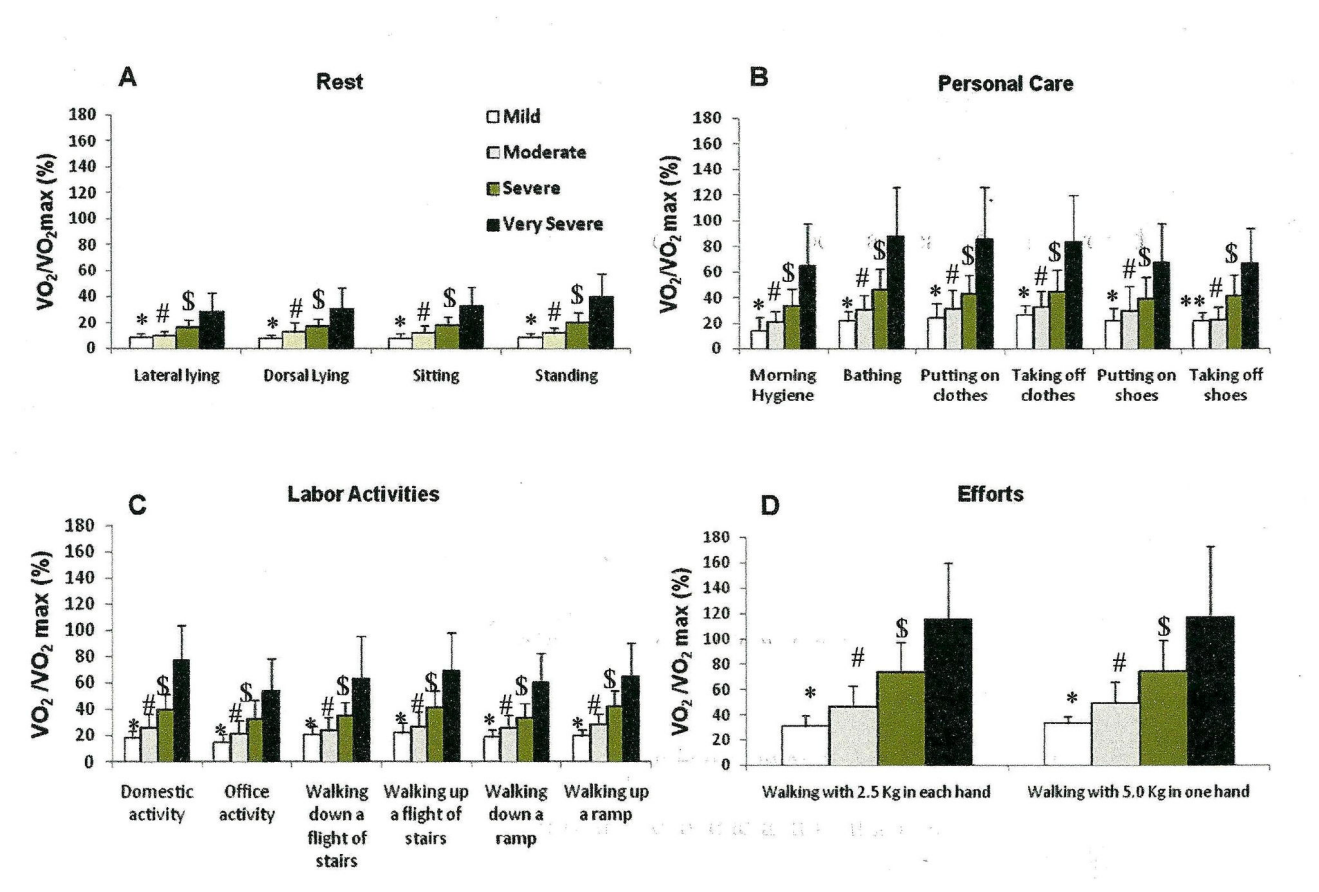
Proportional oxygen consumption/ maximal estimated oxygen consumption (VO_2_/VO_2_max %) ratio at the end of the group of resting activities (A)(lateral and dorsal lying, sitting and standing positions); of personal care (B) (morning hygiene, bathing, putting on and taking off clothes and shoes); of labor activities (C) (domestic and office activities, walking up and down a flight of stairs and ramp); and of efforts activities (D) (walking with 2.5 Kg in each hand and walking with 5.0 Kg in one hand) stratified by the GOLD spirometric classification (mild, moderate, severe and very severe). Mean±standard error. *p<0.01 - mild vs. moderate, severe and very severe; ^#^p<0.01 - moderate vs. severe and very severe; ^$^p<0.05 – severe vs. very severe; ** p<0.01 - mild vs. severe and very severe. Short title: Proportional oxygen consumption/ maximal estimated oxygen consumption (VO_2_/VO_2_max %) ratio after the performed ADL.

**Figure 4:**
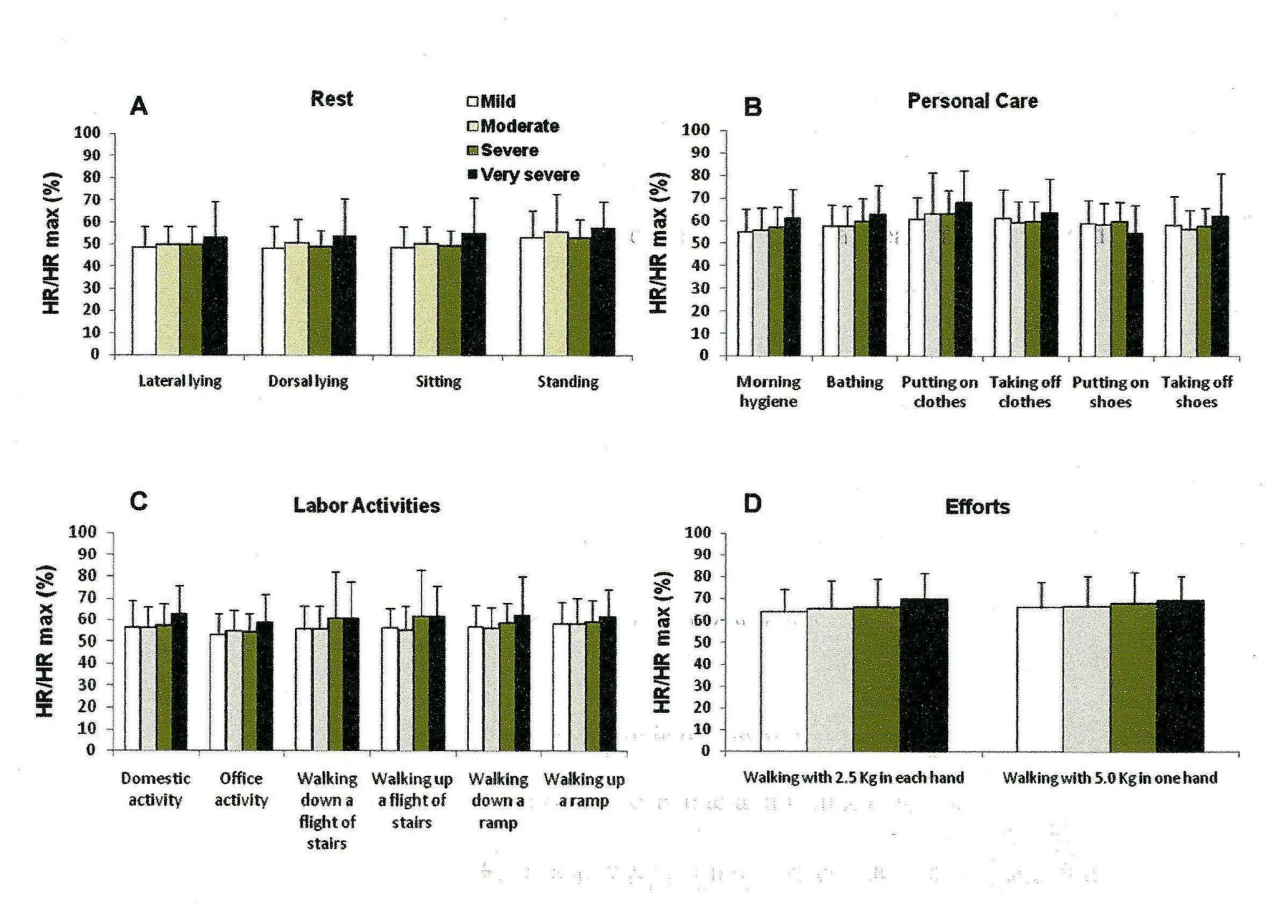
Proportional heart rate/ maximal heart rate (HR/HRmax %) ratio at the end of the group of resting activities (A)(lateral and dorsal lying, sitting and standing positions); of personal care (B) (morning hygiene, bathing, putting on and taking off clothes and shoes); of labor activities (C) (domestic and office activities, walking up and down a flight of stairs and ramp); and of efforts activities (D) (walking with 2.5 Kg in each hand and walking with 5.0 Kg in one hand) stratified by the GOLD spirometric classification (mild, moderate, severe and very severe). Mean±standard error. Short title: Proportional heart rate/ maximal heart rate (HR/HRmax %) ratio after the performed ADL.

**Figure 5:**
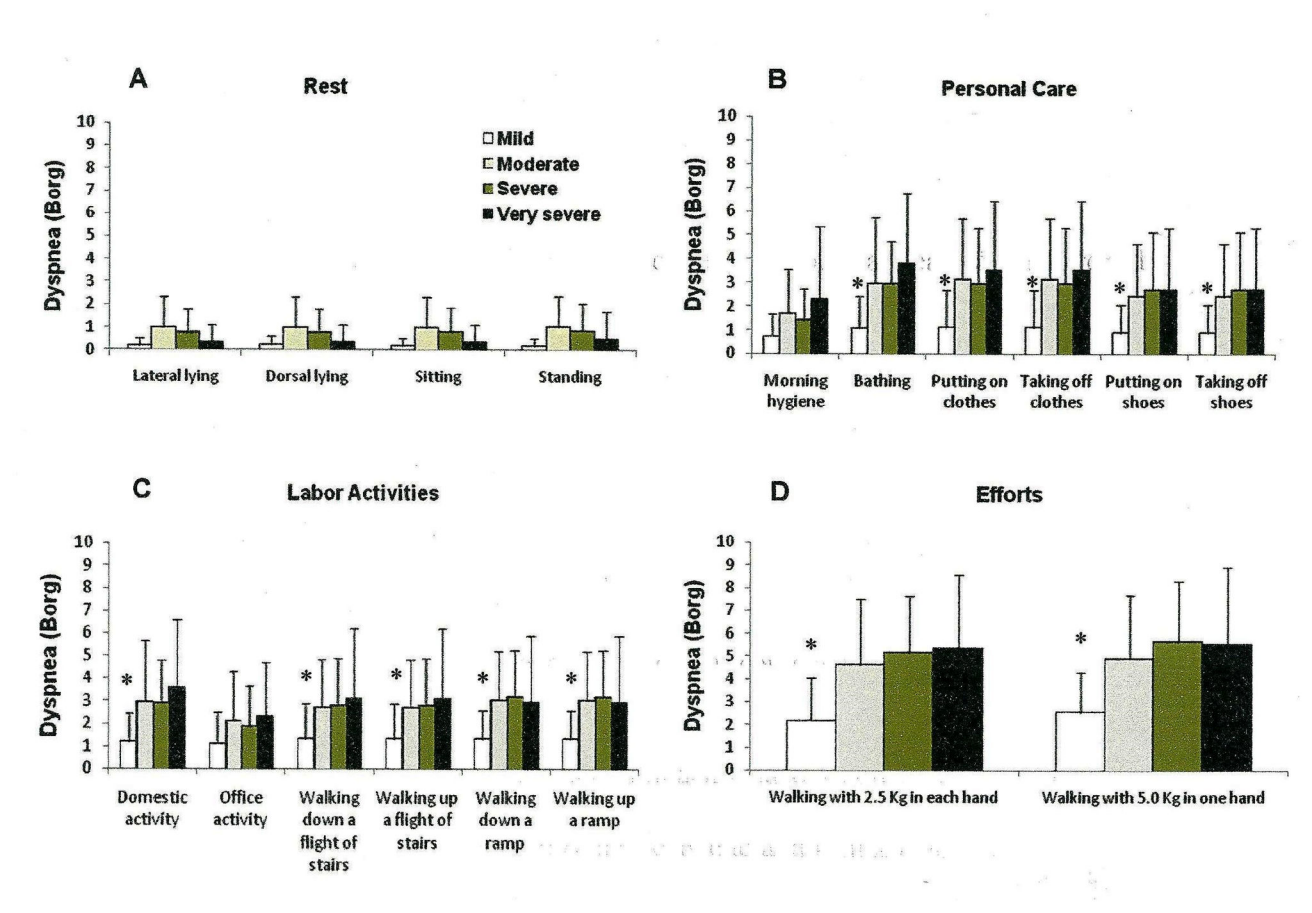
Peak dyspnea at the end of the group of resting activities (A)(lateral and dorsal lying, sitting and standing positions); of personal care (B) (morning hygiene, bathing, putting on and taking off clothes and shoes); of labor activities (C) (domestic and office activities, walking up and down a flight of stairs and ramp); and of efforts activities (D) (walking with 2.5 Kg in each hand and walking with 5.0 Kg in one hand) stratified by the GOLD spirometric classification (mild, moderate, severe and very severe). Mean±standard error. *p<0.01 - mild vs. moderate, severe and very severe. Short title: Peak dyspnea after the performed ADL.

We observed that the more severe the disease, the higher the VE/MVV (%) (Figure 2) and VO_2_/ VO_2_ max (%) (Figure 3). No difference was found among the HR/HRmax (%) across the four different COPD patient groups in any activity (Figure 4). Moderate, severe and very severe patients experienced more dyspnea at the end of the activities of bathing, putting on and taking off clothes and shoes, domestic activity, walking up and down a flight of stairs and a ramp and walking with 2.5 Kg in each hand and 5.0 Kg in just one hand than mild COPD patients (Figure 5). 

We found moderate correlations for dyspnea with VE/MVV (%) at the end of the activities of walking with 2.5 Kg in each hand and 5.0 Kg in just one hand (r=0.53, p=0.01; r=0.43, p=0.01, respectively); and dyspnea with VO_2_/VO_2_max (%) at the end of the same activities (r=0.41, p=0.02; r=0.33, p=0.01, respectively). 

## Discussion

The novel findings of this study in COPD patients are: (1) simple activities of daily living are associated with a high proportion of the estimated peak aerobic capacity; (2) activities performed with movements of legs and arms combined develop the highest ventilatory and oxygen consumption; (3) patients with the most severe disease present the highest proportion of ventilatory and oxygen consumption; (4) activities of daily living are mainly limited by ventilation in COPD patients; (5) COPD patients seem to present no cardiac limitation to complete activities of daily living, regardless of disease severity.

The study was designed taking into consideration two important aspects: (1) activities should be performed as close as possible to “real life”, and (2) they should be reproduced in a very standardized manner. However, “real life” is not the same for all patients. Therefore, we used the video tape in order to normalize the work done by the patients. Our previous experience measuring cost of daily activities in COPD patients [4] showed us that different patients perform the same daily activity in different paces and times. In this case is difficult to know what is the real energy cost of a daily activity. By standardizing the pace and time we can really measure the energy cost of a certain daily activity. But in this case, as expected, the energy cost will be approximately the same independently of the disease severity, as we saw in our patients from mild to very severe disease (Table 2). Obviously, as the disease become more severe the energy reserve gets lower (Figure 3) and patients may get dyspneic when performing some of these activities. 

Thus, the performance time and movement repetitions were closely controlled by using a video tape played on a TV screen in which an actor previously trained executed the exact movements the patients should do. This is the first study to assess such a large number of activities (18) in a very well standardized way, what makes it original. 

Our data shows that the activities that most enhanced ventilatory and oxygen consumption were those related to legs and arms movements combined, such as, bathing, putting on and taking off clothes and carrying 2.5 Kg on each hand and 5.0 Kg on one hand for five minutes. Vaes et. al. [6] evaluated five activities of daily living in COPD patients and also observed that the activities that presented the highest ventilatory and oxygen consumption were those associating upper and lower limbs movements, like sweeping the floor and storing groceries in shelves, what it is in keeping with our results.

Another important outcome of our study is that the greater the disease severity the higher the proportion of ventilatory and estimated peak aerobic capacity, what means a lower ventilatory and aerobic reserve. For instance, domestic activity developed ventilation that corresponded to 19.9% of the VE/MVV in mild COPD patients and 80.6 % in very severe patients; in the same way the estimated aerobic capacity corresponded to 15.1% of VO_2_/VO_2_max in mild COPD patients and 54.0% in very severe patients. These data reinforce the thought that COPD patients should learn and use energy conservation techniques, especially the severe and very severe patients, as they increase the ventilatory and oxygen demands [17]. Velloso et al. [4] assessed severe and very severe COPD patients sweeping the floor and storing bags in high shelves, and found a VE/MVV ratio of 50% and of VO_2_/VO_2_max ratio of 62%, values that are similar to the ones observed in our study. Vaes et al. [6] also showed a VO_2_/VO_2_max ratio of 42% and a VE/MVV of 46% after the sweeping the floor activity in moderate to very severe COPD patients. Likewise, Vaes et al. [6] also observed that the proportion of peak ventilatory and aerobic capacity increases as the severity of the disease progresses. Their data also points out that activities with movements of the arms and legs combined represent an extra burden for these patients.

Dyspnea is an usual limiting factor for COPD patients to perform ADL. Assessment of dyspnea during any intervention is an important information as it is associated to physical capacity [18–21]. We observed that the activities that presented the highest dyspnea scores were bathing, putting on and taking off clothes, domestic activities and walking with 2.5 Kg on each hand and with 5.0 Kg on one hand (Figure 5). Very severe patients usually presented two to four times greater dyspnea scores than mild COPD patients for the same activity. Vaes et al. [6] also showed that GOLD IV COPD patients had higher dyspnea scores for the execution of activities of daily living than COPD GOLD II and III patients. Interestingly and contrary to Vaes et.al. [6], we did not find any difference in the dyspnea scores within the moderate, severe and very severe groups of COPD patients. Nevertheless, their scores were higher than in the mild COPD group. A possible explanation for this difference relies in the fact that the execution of activities were done differently. The patients in our study performed the activity according to a standardized sequence played in the video tape in front of them, while Vaes et. al. [6] asked their patients to perform the activity the way they were used to. The activities that presented the highest VE/MVV (%) like bathing and walking carrying 2.5 Kg on each hand and 5.0 Kg on one hand, were the ones that presented the highest correlations with dyspnea. Dyspnea is a multifactor symptom [22] and has been associated to pulmonary hyperinflation [23].

Hannink et al [24] showed that dynamic pulmonary hyperinflation occurs during the activities of daily living in COPD patients. Likewise, Castro et al [25] showed that COPD patients develop dynamic pulmonary hyperinflation performing simple activities as walking up and down a flight of stairs, walking up and down a ramp and sweeping and mopping the floor. Garcia-Rio et. al. [26] observed that 89 out of their 110 moderate and severe COPD patients developed dynamic pulmonary hyperinflation during activities of daily living.

We found no difference between the HR/HRmax ratio within the four disease stages at the end of the activities (Figure 4). The cardiac reserve at the end of the activities clearly shows that no heart limitation occurred. Vaes et. al. [6] also showed that their COPD patients presented lower HR/HRmax (%) and higher VE/MVM (%) than a healthy elderly control group, showing that their patients had ventilatory but no cardiac limitation at the end of the activities performed.

We may consider some limitations in our study. The lack of a control group may be considered only a partial limitation, as there are already enough published literature that reports normal subjects ventilatory and metabolic response during activities of daily living [4,6]. Therefore, we did not expect that this information would bring any additional data to the literature.

Another limitation might be the fact that peak VO_2_ was estimated and not measured. However, there are plenty of equations for VO_2_ prediction in COPD patients [3,4,12,13] and we decided not to measure the VO_2_max because we did not want to cause an extra stress to our patients such as to submit them to a CPET. In addition, we chose an equation to estimate the VO_2_max that is widely accepted [3]. The activities of daily living were performed only once because it has been shown before that they are very reproducible [4,6]. 

Due to the use of the facial mask, patients had to mimic, and not actually perform some activities (e.g., washing the face, brushing teeth and combing hair). Despite the fact that this was a limitation of the procedure, we believe that it did not influence the final outcome, once the movements were carried out exactly as they are performed in “real life”. Another possible limitation in our study is that the 18 activities were performed in a sequence what it is not usual in real life, and it may be presumed that a possible additional ventilatory and oxygen consumption left from the previous activity that would overestimate the ventilatory and oxygen consumption of the following activity. However, patients were allowed to rest before each activity in order for the ventilatory and metabolic level to return to baseline. And finally, some activities were completed in less than five minutes, which is the minimum period of time expected for the ventilatory and metabolic demands to reach the steady-state. However, we intentionally chose to maintain some activities without a fixed performance time so they could really simulate it as they are performed in “”real life”.

The clinical implication of our study is: that knowing the ventilatory and metabolic burden an activity demands would help the health care team to conduct the management of COPD patients. For instance patients could be better educated and encouraged to use energy conservation techniques and the rehabilitation program could be focused on the activities the patient is more limited. 

Finally, the originality of this study that it was the first one to evaluate such a large number of activities of daily living, besides being a very standardized manner, and with all four COPD patients stages. The activities tested in our study are the ones commonly performed throughout the day by COPD patients.

 This study has allowed us to conclude that: COPD patients performing several activities of daily living reach an increased proportion of the estimated peak ventilation and aerobic capacity; besides, the activities that develop the higher proportion of the ventilatory and aerobic consumption are the ones associated to upper and lower limbs movements combined; very severe patients present the highest proportion of ventilatory and aerobic consumption and dyspnea; the execution of activities of daily living are mainly limited by COPD’s reduced ventilatory reserve; no cardiac limitation appears to influence the completion of ADLs by COPD patients, regardless of disease severity. We expect that future studies would validate this table as a tool for teaching COPD patients the use of energy conservation techniques. 
